# Management of low-grade squamous intraepithelial lesions of the uterine cervix

**DOI:** 10.1038/sj.bjc.6601415

**Published:** 2004-03-02

**Authors:** C Scheungraber, N Kleekamp, A Schneider

**Affiliations:** 1 Department of Obstetrics and Gynecology, Friedrich Schiller University, Jena, Germany; 2 Computing Center, Friedrich Schiller University, Jena, Germany

**Keywords:** LSIL, management LSIL, cervical lesions

## Abstract

Strategies of management for low-grade squamous intraepithelial lesion (SIL) vary even on a national level. We evaluated the diversity of management algorithms. This should serve as a source to find a common basis for the management of low-grade SIL. A total of 38 representatives and specialists for colposcopy and cervical pathology were contacted to provide national guidelines, recommendations or consensus for the management of patients diagnosed with the cytologic diagnosis of low-grade SIL. In all, 23 addressees (60%) responded. The algorithms provided varied considerably. Three variants of algorithms could be defined. Variant 1 was proposed in 14 countries and recommended colposcopy immediately after cytologic diagnosis of low-grade SIL or at the same time the smear is taken. If available, HPV testing was recommended as a triage option in some countries. Variant 2 is used in three countries and colposcopy is only performed after a repeated abnormal cytologic result within a 6-month interval or after an optional test positive for high-risk HPV. Variant 3, as proposed in six countries, takes into account the socio-economic status of the patient: In patients with poor compliance, ‘see and treat’ management is recommended; in patients where compliance can be assured, follow-up is carried out by cytology and colposcopy. Global policy of management of patients with low-grade SIL can be summarised in three algorithms. Quality standards and outcome parameters must be defined in order to improve the management of women with low-grade SIL.

Low-grade squamous intraepithelial lesion (SIL) has been defined as a cytologic diagnosis for patients with smears showing cytologic criteria of permissive HPV infection or CIN I (Anonymous, 1989). This classification (Richart, 1990) is also used for histopathologic diagnosis, and low-grade SIL and low-grade CIN are used synonymously. The gold standard for the definition of cervical disease is histopathologic evaluation. However, it has to be kept in mind that even between expert pathologists inter- and intraobserver variability is high: in a recent study on 194 cervical tissues of different histologic severity, the agreement rate between five expert pathologists was 52% for the diagnosis of CIN I (*κ* value 0.6) (Klaes *et al*, 2002). Agreement could be improved to 91% (*κ* value 0.9) by immunohistochemical staining for p16^ink4a^ a marker associated with the presence of high-risk HPVs.

In addition to the problem of defining the disease accurately, the natural course of low-grade SIL is variable: The majority of lesions (up to 70%) regress and only 10% of low-grade SIL may progress to high-grade CIN (Nasiell *et al*, 1986; Östor, 1993; Holowaty *et al*, 1999). When patients with the cytologic diagnosis of low-grade SIL are stratified in high-risk HPV negative or high-risk HPV positive using a GP5+ GP6+ system, all high-risk HPV-negative and 70% of high-risk HPV-positive smears become normal over a period of 4 years (Nobbenhuis *et al*, 2001). In this series, 62% of smears were high-risk HPV positive. Not a single patient out of 64 patients with the cytologic diagnosis of low-grade SIL progressed to high-grade CIN (Nobbenhuis *et al*, 1999). However, using the only commercially available HPV test in clinical practice, Hybrid Capture II triaging low-grade SIL in regressors and progressors is not recommended due to the high rate of high-risk HPV positives with this system (i.e. 83%) (Koutsky *et al*, 1992; Stoler, 2001).

Since conventional histopathologic evaluation and HPV testing do not allow to define the biologic potential of low-grade SIL, there is a wide variety of clinical recommendations for management of disease. It was the purpose of this study to gain an overview of the current clinical practice in various countries in order to see if there is a need for simplification and coordination, for integration of new markers, and a basis for defining common quality and outcome parameters for the management of women with low-grade SIL.

## MATERIALS AND METHODS

A letter was sent to 38 different societies and institutions responsible for the definition and coordination of management of patients with cervical abnormalities in the various countries. If published memoranda, recommendations, guidelines or consensus statements or clinical pathways were available, the authors were asked to provide the references or to send copies of the published material. If no such written statements were available, the authors were asked to give their personal interpretation of the national strategies. For each country, an algorithm was produced and algorithms that had only minor discrepancies were combined. Collection of algorithms was carried out between July and September 2002 and interpretation of algorithms was presented at the 20th International Papillomavirus Conference in Paris from 4 to 9 October 2002.

## RESULTS

**Figure 1:**
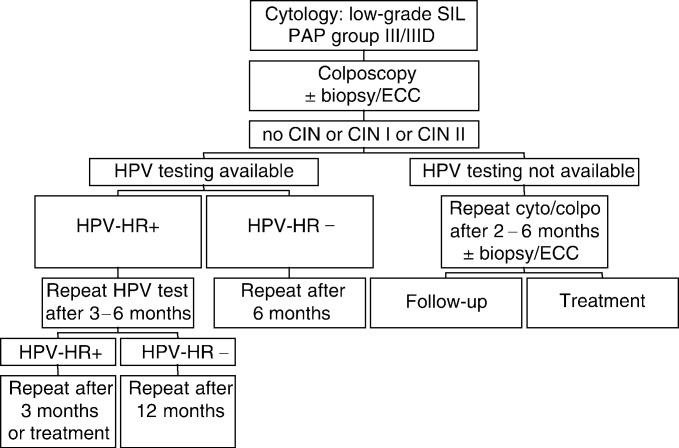
Algorithm 1: colposcopy immediately following cytologic diagnosis of low-grade SIL or in each check-up as a routine examination described for Austria (Girardi *et al*, 2001), Germany, Greece, Hong Kong (Ngam *et al*, 1999), Hungary, Israel, Italy, Poland, Portugal, Russia, Spain, Sweden (Stockholm area), USA (Wright *et al*, 2002), Yugoslavia (Kesic, 2002). This is not the algorithm of the American Society of Colposcopy and Cervical Pathology (ASCCP), which can be viewed at www.asccp.org.

**Figure 2:**
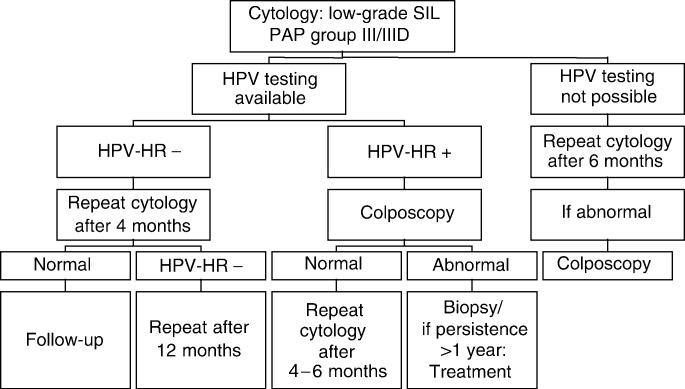
Algorithm 2: repeat cytology following cytologic diagnosis of low-grade SIL described for Croatia (Ljubojeviæ *et al*, 2002), England (Duncan, 1997), New Zealand.

Colposcopy immediately after the cytologic diagnosis of low-grade SIL is recommended in 11 countries (algorithm 1, Figures 1

and 4). In three European countries (Hungary, Spain and Yugoslavia) – where colposcopy is used as part of routine gynaecologic examination – colposcopy is also considered as an essential tool for managing low-grade SIL, but the colposcopic examination is carried out at the time the smear is taken. In Sweden, there are two different policies for various regions: When a low-grade lesion is detected in a routine cytologic smear, women in the Stockholm area are referred for colposcopy within 4 months. In other parts of Sweden, women with the cytologic diagnosis of low-grade SIL are followed up according to algorithm 2 (Figures 2

and 4).

Following histologic diagnosis of low-grade SIL after 2–6 months repeat examination by cytology and colposcopy is performed in all these countries. Persistent low-grade SIL can be either followed or treated. In health-care systems where HPV testing is available and recommended, high-risk HPV positive patients are followed up closely. In the US, HPV-testing is acceptable not initially, but after 12 months (please see www.asccp.org).

In four countries, cytologic diagnosis of low-grade SIL is followed up by a repeat smear after 6 months and only, when repeatedly abnormal on cytology, colposcopy is performed. When HPV testing is available, high-risk HPV-positive patients undergo colposcopy and, when abnormal findings are confirmed and persist for more than 1 year, treatment is performed (algorithm 2, Figures 2 and 4).

**Figure 3:**
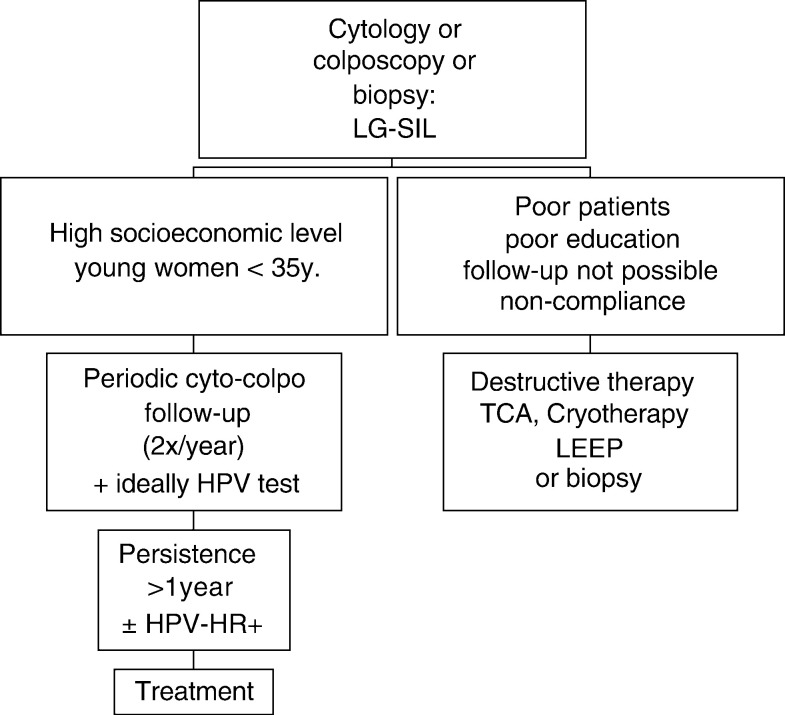
Algorithm 3: management of patients with the cytologic diagnosis of low-grade SIL according to socio-economic status described for Argentina, Chile, India, Paraguay, Philippines, Slovakia.

In six countries, management is performed according to the socio-economic status (algorithm 3, Figures 3

and 4

**Figure 4:**
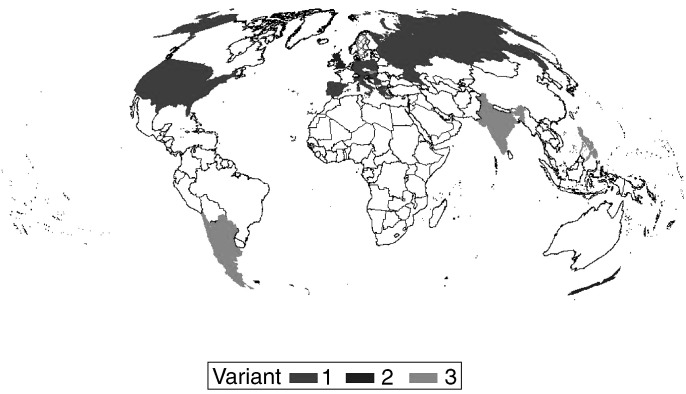
World map: Black: countries following algorithm 1; hatched: Sweden Stockholm area following algorithm 1, other parts following algorithm 2. Grey: countries following algorithm 2. Chequered: countries following algorithm 3.

). If noncompliance can be expected, ‘see and treat’ with different treatment modalities is recommended. In patients with high compliance, periodic evaluation by cytology and colposcopy is recommended and can be augmented by HPV testing. Lesions persisting for more than 1 year should be treated.

## DISCUSSION

Management of women with the cytologic diagnosis of low-grade SIL varies considerably according to the availability of medical resources and socio-economic status of women. Since the histopathologic basis of disease is ill defined and progression markers are not available, there is a need for standardisation of treatment in order to guide the patient and clinician to avoid over- or undertreatment.

Immediate colposcopy after cytologic diagnosis of low-grade SIL (algorithm 1) is costly and may cause unnecessary stress on patients and the health system in countries where colposcopy is not a routine part of the gynaecologic examination. However, delay of histopathologic confirmation by 6 months or longer (algorithm 2) may lead to underdiagnosis and worsening of prognosis. For Germany, it has been shown that in patients with the cytologic diagnosis of low-grade SIL, CIN III is prevalent in 30.2% and invasive cancer in 1% of patients (Petry *et al*, 1999). In countries with excellent quality control of cytology, this rate may be lower and cytologic follow-up may be regarded sufficient.

In health-care systems where high-risk HPV testing is available, triaging in high-risk HPV-positive and high-risk HPV-negative lesions is performed (algorithms 1, 2, and 3). However, for clinical practice, no clear guidelines have been established as to testing of smears or biopsies and choice of HPV test. Tests that include only a certain amount of pooled high-risk HPVs and that show crossreaction between high- and low-risk types are clinically not useful due to low specificity (Arbyn, 2001). Highly sensitive two-tier PCR systems including a panel of 50 HPV types applied to tissue sections and not to smears may be more specific: only 30% of 453 CIN I contained high-risk HPV types and prevalence and distribution of HPV types are significantly different from CIN II or CIN III (Feoli-Fonseca *et al*, 2001). Thus, HPV systems with improved sensitivity and specificity may become clinically useful. Persistent detection of high-risk HPV, integration of high-risk HPV DNA in the host cell DNA, high viral load, and/or presence of certain HPV variants may be additional markers for future use. ‘See and treat’ as recommended by various countries (algorithm 3, Figure 3) on the basis of low-grade SIL by cytology alone, without biopsy-confirmed CIN I or more severe lesions, is not recommended in the US (please see www.asccp.org) (Figure 4).

There is a need to coordinate the diversing algorithms for the management of patients with low-grade SIL in order to avoid overtreatment: The majority of women with diagnosis of low-grade SIL are young and still desire conception. Standard treatment is still invasive, either using excisional or ablative procedures, which are associated with a peri- and postoperative morbidity rate of up to 10%. The data presented in this enquiry should therefore serve as a basis to start a discussion of a coordinated effort to improve the quality and result of the management of this highly prevalent diagnosis in order to improve women's health.

## Appendix

### GROUP OF SPECIALISTS

Basta, Antoni: Polish Society of Colposcopy and Cervical Physiopathology, Chair and Department of Gynecology and Oncology, Jagellonian University, 31-501 Kopernika Street, Krakow 31501, Poland.

Bornstein, Jacob: Israelian Society of Cervical Pathology and Colposcopy, Department of Obstetrics and Gynecology, Carmel Medical Center, 7 Michal St, Haifa 34 362, Israel.

Boselli, Fausto: Chief of the Oncologic and Preventive Gynaecology Unit, Department of Obstetrics and Gynaecology, University of Modena and Reggio Emilia, Via Del Pozzo 71, 41 100 Modena, Italy.

Bosze, Peter: Hungarian Society of Cervical Pathology and Colposcopy, Saint Stephen Hospital, Nagyvarad ter. 1, 1096 Budapest, Hungary.

Chase, Luis Antonio: Paraguay Society of Pathology of the Lower Genital Tract and Colposcopy, Martin Brizuela 750c/Lillo, Asunción, Paraguay.

Clentworth, Howard: President New Zealand Society of Colposcopy and Cervical Pathology, Women's Health Service, Capital Coast Health Bd., Private Bay 7902, Wellington, New Zealand.

Cortes-Bordoy, Javier (Montserrat Cararach Tur): Spanish Society of Cervical Pathology and Colposcopy, Clinica Serosa, Sor Francinaina Cirer, 1, 07011 Palma, España.

Das, SK: Chief of Gynae Oncology, Rajiv Gandhi Cancer Institute & Research Centre, Sector-5, Rohini, Delhi 110 085, India.

Diakomanolis, Emmanuel: Hellenic Society of Cervical Pathology (Greece), Colposcopy, and Laser, Athens University, 82 Vasilissis Sofias Ave., 11528 Athens, Greece.

Fai, Tam Kar: Secretary General, Hong Kong Society for Colposcopy and Cervical Pathology, OB/GYN, Queen Mary Hospital, 102 Pokfulam Rd., Hong Kong.

Girardi, F (Girardi, F, Pickel, H, Joura, EA, Breitenecker, G, Gitsch, G, Graf, A-H, Neunteufel, W): Abteilung für Gynäkologie und Geburtshilfe, Allgemein-öffentliches Krankenhaus der Kurstadt Baden, Wimmergasse 19, 2500 Baden, Österreich (Austria).

Grubišiæ, Goran (Ljubojeviæ, N, Babiæ, S, Audy-Jurkoviæ, S, Ovanin-Rakiæ, A, Grubišiæ, G, and Ljubojeviæ-Grgec, D): Head of Conservative Gynecology Department at University Hospital ‘Sisters of Mercy’ and President of the Croatian Society for Colposcopy and Cervical Pathology, 10 000 Zagreb, Vinogradska 29, Croatia.

Heinrich, Juergen: German Federation of Cervical Pathology and Colposcopy, Obstetrics and Gynecology, Klinikum der Hansestadt Stralsund GmbH, Grosse Parower Street 47-53, 18435 Stralsund, Germany.

Kesic, Vesna: Yugoslav Society of Cervical Pathology and Colposcopy, Institute of Obstetrics and Gynecology, University Clinical Center, 11 000 Belgrad, Yugoslavia.

Kitchener, Henry: British Society of Cervical Pathology and Colposcopy, Department of Obstetrics and Gynecology, St Mary's Hospital, Whitworth Park, Manchester, UK.

Limson, Genara A Manuel: Philippines Society of Cervical Pathology and Colposcopy, Makati Med. Ctr., 2 Amorsolo St., Mahati City, Philippines.

Morales, Eduardo: Chilean Society of Cervical Pathology and Colposcopy (Sociedad Chilena de Colposcopia) Santiago, Chile.

Peluffo, Marcos: Argentinian Society of Cervical Pathology and Colposcopy, Posadas 1567 PB A (C1112ADA), Buenos Aires, Argentina.

Pereira da Silva, Daniel: Portugal Society of Colposcopy and Cervical Pathology, Ty Dr Manuel Bastos Pina 58, 3000 Coimbra, Portugal.

Rylander, Eva: Karolonska Institutet Danderyds Hospital, Deputy Head of Department (Division of Obstetrics and Gynaecology), SE-18288 Stockholm, Sweden.

Singer, Albert: Whittington Hospital, Department of Women's & Children's Health, Jenner Building, Highgate Hill, London N19 5NF, UK.

Štefanoviè, Julius: Slovak Society of Cervical Pathology and Colposcopy, National Cancer Institute (Národnýy Onkologický U̇stav), Department of Gynecology, Klenová 1, 81310 Bratislava, Slovak Republic.

Wilkinson, Edward J (Wright Jr, TC, Cox, JT, Massad, S, Twiggs, LB, and Wilkinson, EJ): Past President American Society of Cervical Pathology and Colposcopy, 20 W. Washington St., Suite 1, Hagerstown, MD 21740, USA.

### GUIDELINES

Duncan I (ed.). Guidelines for clinical practice and programme management. NHSCSP 1997, No. 8.

Girardi, F, Pickel, H, Joura, EA, Breitenecker, G, Gitsch, G, Graf, A-H, Neunteufel, W. Leitlinien für Diagnose und Therapie intraepithelialer Neoplasien und frühinvasiver Karzinome des unteren Genitaltraktes der Frau (Cervix uteri, Vagina, Vulva), erstellt von der AGK (Arbeitsgemeinschaft für Kolposkopie in der ÖGGG). Gynäkol Geburtshilfliche Rundsch 2001; 41: 197–200.

Kesic V. Guidelines for management of abnormal cervical findings – Yugoslav Society for Colposcopy and Cervical Pathology.

Ljubojeviæ, N, Babiæ, S, Audy-Jurkoviæ, S, Grubišiæ, G, Ljubojeviæ-Grgec, D. Diagnostic and therapeutic guideline in cytological diagnosis of cervical glandular atypia (AGCUS, GIL I, GIL II, and AIS).

Ljubojeviæ, N, Babiæ, S, Audy-Jurkoviæ, S, Ovanin-Rakiæ, A, Grubišiæ, G, Ljubojeviæ-Grgec, D. Diagnostic and therapeutic guideline in cytologic diagnosis of cervical atypia (ASCUS, CIN I–III).

Ngam H, Ng T, Collins RJ, Cheung A, So LK, Fan S, Chan M. Guidelines on the management of an abnormal cervical smear. HKCOG Guidelines 1999, No. 3

Wright TC, Cox JT, Massad LS, Twiggs LB, Wilkinson EJ for the 2001 ASCCP-sponsored Consensus Conference, 2001. Consensus Guidelines for the management of women with cervical cytological abnormalities. JAMA 2002; 287: 2120–2129.

Wright TC, Cox JT, Massad LS, Twiggs LB, Wilkinson EJ for the 2001 ASCCP-sponsored Consensus Conference. Algorithms from the Consensus Guidelines for the management of women with cervical cytological abnormalities, ASCCP, www.asccp.org.
